# Human Scavenger Receptor A1-Mediated Inflammatory Response to Silica Particle Exposure Is Size Specific

**DOI:** 10.3389/fimmu.2017.00379

**Published:** 2017-04-03

**Authors:** Nobuo Nishijima, Toshiro Hirai, Kazuki Misato, Michihiko Aoyama, Etsushi Kuroda, Ken J. Ishii, Kazuma Higashisaka, Yasuo Yoshioka, Yasuo Tsutsumi

**Affiliations:** ^1^Laboratory of Toxicology and Safety Science, Graduate School of Pharmaceutical Sciences, Osaka University, Suita, Japan; ^2^Vaccine Creation Project, BIKEN Innovative Vaccine Research Alliance Laboratories, Research Institute for Microbial Diseases, Osaka University, Suita, Japan; ^3^Laboratory of Vaccine Science, WPI Immunology Frontier Research Center (iFReC), Osaka University, Suita, Japan; ^4^Laboratory of Adjuvant Innovation, National Institutes of Biomedical Innovation, Health and Nutrition (NIBIOHN), Ibaraki, Japan; ^5^BIKEN Center for Innovative Vaccine Research and Development, The Research Foundation for Microbial Diseases of Osaka University, Suita, Japan; ^6^The Center for Advanced Medical Engineering and Informatics, Osaka University, Suita, Japan

**Keywords:** inflammasome, Mer receptor tyrosine kinase, nanoparticles, nanotoxicity, scavenger receptor, size

## Abstract

The application of nanotechnology in the health care setting has many potential benefits; however, our understanding of the interactions between nanoparticles and our immune system remains incomplete. Although many of the biological effects of nanoparticles are negatively correlated with particle size, some are clearly size specific and the mechanisms underlying these size-specific biological effects remain unknown. Here, we examined the pro-inflammatory effects of silica particles in THP-1 cells with respect to particle size; a large overall size range with narrow intervals between particle diameters (particle diameter: 10, 30, 50, 70, 100, 300, and 1,000 nm) was used. Secretion of the pro-inflammatory cytokines interleukin (IL)-1β and tumor necrosis factor (TNF)-α induced by exposure to the silica particles had a bell-shaped distribution, where the maximal secretion was induced by silica nanoparticles with a diameter of 50 nm and particles with smaller or larger diameters had progressively less effect. We found that blockade of IL-1β secretion markedly inhibited TNF-α secretion, suggesting that IL-1β is upstream of TNF-α in the inflammatory cascade induced by exposure to silica particles, and that the induction of IL-1β secretion was dependent on both the NLRP3 inflammasome and on uptake of the silica particles into the cells *via* endocytosis. However, a quantitative analysis of silica particle uptake showed that IL-1β secretion was not correlated with the amount of silica particles taken up by the cells. Further investigation revealed that the induction of IL-1β secretion and uptake of silica nanoparticles with diameters of 50 or 100 nm, but not of 10 or 1,000 nm, was dependent on scavenger receptor (SR) A1. In addition, of the silica particles examined, only those with a diameter of 50 nm induced strong IL-1β secretion *via* activation of Mer receptor tyrosine kinase, a signal mediator of SR A1. Together, our results suggest that the SR A1-mediated pro-inflammatory response is dependent on ligand size and that both SR A1-mediated endocytosis and receptor-mediated signaling are required to produce the maximal pro-inflammatory response to exposure to silica particles.

## Introduction

The application of nanotechnology is a promising means of developing novel diagnostic and imaging technologies, photothermal therapies, vaccines, and drug delivery systems (1–3). However, various immune toxicities associated with exposure to nanoparticles have been reported, including inflammation (4), immune suppression (5), IgE-biased immune responses (6), and the induction of metal allergies (7). Therefore, improving our understanding of the interactions between nanoparticles and our immune system is essential to ensure the safe use of nanotechnology in the health care setting.

There are two main factors that make nanoparticles not only more effective but also more hazardous than the bulk material. The first is their ability to cross biological barriers [e.g., blood–brain barrier (8), placental barrier (9), blood–milk barrier (10), and nuclear barrier (11)]. The second is their large surface area per unit mass due to their small particle size. Since biological interactions occur on the surface of nanoparticles, the biological activity of nanoparticles per unit mass increases as particle size decreases (12). Indeed, many studies, both *in vitro* and *in vivo*, have demonstrated that smaller nanoparticles have biological activities of greater strength compared with larger particles (13–16). However, several *in vitro* studies have also shown that nanoparticles with a diameter of 50 nm are more readily taken up by cells and/or have greater cytotoxicity than larger and smaller particles of the same material (17–20). Indeed, we recently identified a size-specific effect in mice, where silica nanoparticles with a diameter of 50 nm induced the most severe hypothermia in the 10–1,000 nm size range (21). In addition, it has been reported that in a comparison of nanoparticle-based antitumor vaccines that differed only with respect to particle diameter (20, 40, 100, 200, 500, 1,000, or 2,000 nm), the vaccine with a particle diameter of 40 nm was the most effective (22). Together, these studies demonstrate not only that size-specific biological effects of nanoparticles exist but also that particles with diameters of around 50 nm induce the strongest biological effects. Further studies are needed to elucidate the mechanisms underlying these size-specific effects.

The pro-inflammatory effects of nanoparticles are well described in the literature and are a major issue for the development of safe nanomedicines (23). In particular, the NLRP3 inflammasome-mediated pro-inflammatory effects of nanoparticles have been reported (4, 24–26). However, the effect of particle size on the pro-inflammatory effects of nanoparticles is poorly understood, most likely because previous studies did not examine a particle size range that included fine enough intervals between particle sizes.

In the present study, we examined the effects of particle size on the pro-inflammatory response of THP-1 cells to exposure to silica particles within a large overall size range (10–1,000 nm) that included narrow intervals between the particle diameters. We also explored the mechanisms underlying this size-specific inflammatory response in our model, although it should be noted that the experimental conditions were not chosen to represent human exposure scenarios.

## Materials and Methods

### Silica Particles

Amorphous silica particles (silica particles) with diameters of 10, 30, 50, 70, 100, 300, or 1,000 nm (nSP10, nSP30, nSP50, nSP70, nSP100, mSP300, and mSP1000, respectively) were purchased from Micromod Partikeltechnologie (Rostock/Warnemünde, Germany). Crystalline silica particles (Min-U-Sil-5; crystalline silica in diameter of not bigger than 5 μm) were purchased from Pennsylvania Sand Glass Corporation (Pittsburgh, PA, USA). The endotoxin level of each size of silica particle (50 μg/mL in cell culture media) was 0.25, 0.15, 0.11, 14.88, 1.23, 0.01, and <0.01 endotoxin units/mL for nSP10, nSP30, nSP50, nSP70, nSP100, mSP300, and mSP1000, respectively, as determined by a Pyros Kinetix turbidity assay instrument with a limit of detection of 0.001 endotoxin units/mL. Endotoxin testing was performed on our behalf by nanoComposix (San Diego, CA, USA). Immediately prior to use, the dispersions of the particles were sonicated at 400 W for 5 min at 25°C and then vortexed for 1 min.

### Reagents

Phorbol 12-myristate 13-acetate (PMA), polyinosinic acid potassium salt (poly I), cytochalasin D, bafilomycin A1, BMS345541, and adenosine 5′-triphosphate disodium salt hydrate (ATP) were purchased from Sigma Aldrich (St. Louis, MO, USA). zYVAD-fmk and UNC569 were purchased from Merck (Darmstadt, Germany).

### THP-1 Cells

THP-1 cells (human acute monocytic leukemia cell line) were obtained from the American Type Culture Collection (Manassas, VA, USA) and cultured at 37°C (95% room air, 5% CO_2_) in RPMI1640 (Wako Pure Chemical Industries, Osaka, Japan) supplemented with 10% fetal bovine serum, 1% antibiotic cocktail (10,000 U/mL penicillin, 10,000 μg/mL streptomycin, and 25 μg/mL amphotericin B; Gibco, BRL, Bethesda, MD, USA), and 2-mercaptoethanol (50 μM; Gibco).

### Evaluation of the Pro-inflammatory Activity of the Silica Particles

THP-1 cells (3.0 × 10^4^ cells/well) were seeded in flat-bottom 96-well plates (Nunc, Rochester, NY, USA) and then differentiated into macrophages by incubation with 0.5 μM PMA at 37°C for 24 h. After incubation, the cells were washed with the cell culture media and treated with the silica particles, crystalline silica, or ATP. After incubation for 6, 12, or 24 h, the supernatants were collected. To determine cell viability after exposure to the test materials, the concentration of lactate dehydrogenase in the supernatants was measured by using a Cytotoxicity LDH Assay Kit (Wako, Osaka, Japan) in accordance with the manufacturer’s instructions. To evaluate the pro-inflammatory response to exposure to the test materials, the concentrations of the pro-inflammatory cytokines interleukin (IL)-1β and tumor necrosis factor (TNF)-α, and of the receptor antagonist (RA) IL-1RA, in the supernatants were assessed by ELISA kits (IL-1β, BD Pharmingen, San Diego, CA, USA; TNF-α, eBioscience, San Diego, CA, USA; IL-1RA, R&D Systems, Minneapolis, MN, USA) in accordance with the manufacturers’ instructions. In inhibitory and neutralizing antibody assays, cytochalasin D, zYVAD-fmk, BMS345541, bafilomycin A1, anti-human scavenger receptor (SR) A1 monoclonal antibody (351620) (R&D Systems) or its mouse IgG1 isotype control (BioLegend, San Diego, CA, USA), anti-human macrophage receptor with collagenous structure (MARCO) antibody (PLK1) (Hycult Biotech, Uden, The Netherlands) (27) or its mouse IgG3 isotype control (BioLegend), recombinant human IL-1RA (R&D systems), or anti-human IL-1β/IL-1F2 (2805) (R&D systems) were added to the wells containing the PMA-differentiated THP-1 cells 30 min before stimulation with the test materials.

### Western Blotting Analysis

THP-1 cells (9.0 × 10^5^ cells/well) were seeded in 6-well plates (Nunc) and then differentiated into macrophages by incubation with 0.5 μM PMA at 37°C for 24 h. After incubation, the cells were washed with the cell culture media and treated with the silica particles (50 μg/mL), crystalline silica (500 μg/mL), or ATP (3 mM). After incubation for 6, 12, or 24 h, the cells were washed twice with phosphate-buffered saline and lysed with Mammalian Protein Extraction Reagent (M-PER; Thermo Fisher Scientific, Rockford, IL, USA). Protein samples (1 μg) were loaded on a 20% sodium dodecyl sulfate–polyacrylamide gel. After electrophoresis, proteins were transferred to polyvinylidene difluoride membranes (GE Healthcare, Buckinghamshire, UK). The blots were blocked with 1% BSA in phosphate-buffered saline with 0.02% Tween 20 for 2 h at room temperature. The blots were incubated with monoclonal antibody to human IL-1β/IL-1F2 (8516) (R&D systems) at 1 h. HRP-conjugated goat anti-mouse antibody (SouthernBiotech, Birmingham, AL, USA) was added to the membranes, which were then incubated for 1 h at room temperature. The protein bands on the membrane were visualized with SuperSignal West Femto Maximum Sensitivity Substrate (Thermo Fisher Scientific), and the images were captured by LAS4000 mini (GE Healthcare). The densities of the bands in the captured image were analyzed by using the ImageJ software (version 1.46r, National Institutes of Health).

### Inductively Coupled Plasma Atomic Emission Spectrometry (ICP-AES) Analysis

THP-1 cells (1.4 × 10^7^ cells/dish) were seeded in 150-mm dishes and differentiated into macrophages by incubation with 0.5 μM PMA at 37°C for 24 h. After incubation, the cells were washed with phosphate-buffered saline and incubated with 50 μg/mL of each test material for 6, 12, or 24 h. In a neutralization assay, PMA-differentiated THP-1 cells were pre-incubated for 30 min with anti-human SR-A1 or its isotype control at a concentration of 0.4 μg/mL. After incubation with the test materials, the supernatant was removed and the cells were washed twice with phosphate-buffered saline. The cells were then detached from the dish surface using trypsin, washed with the cell culture media, and collected. After the cells were collected, samples from three dishes were pooled for analysis. The pooled cells were counted, suspended in 1 mL of MilliQ water, and sent to Japan Food Research Laboratories (Osaka, Japan), where the samples were prepared for ICP-AES analysis as follows: the cells were heated to 500°C and ash melted with sodium carbonate. Water was added to the residue and the mixture was heated for 30 min before being passed through filter paper. The filtrates were then brought to a volume of 50 mL with ultrapure water. The mass of silicon in each sample was then measured with a Vista-MPX ICP-AES instrument (Varian, Palo Alto, CA, USA) on our behalf by Kiyokawa Plating Industry Co., Ltd. (Fukui, Japan). Silicon uptake by the cells was calculated as the amount of silicon in silica particle-treated cells minus the silicon level in non-silica-treated cells.

### Statistical Analyses

Statistical analyses were performed by using the Ekuseru-Toukei 2012 software (Social Survey Research Information Co., Ltd., Tokyo, Japan). Data are presented as mean ± SD. Significant differences between the control group and experimental group were assessed by using Student’s *t*-test. *P* < 0.05 was considered statistically significant.

Methods used in the Supplementary Figures are in the supplementary figures file.

## Results

### Effect of Particle Size on the Pro-inflammatory Effect of Silica Particles in THP-1 Cells

The hydrodynamic diameters of the silica particles dispersed in the cell culture medium (5 mg/mL), as measured by means of dynamic light scattering, were 10.0, 24.3, 48.3, 64.7, 86.0, 285.7, and 1,164.3 nm for nSP10, nSP30, nSP50, nSP70, nSP100, mSP300, and mSP1000, respectively (Table S1 in Supplementary Material). These hydrodynamic diameters suggest that the silica particles were well dispersed in the cell culture medium. Transmission electron microscopy images of the silica particles used in the present study are provided in our previous reports (6, 21, 28).

**Figure 1:**
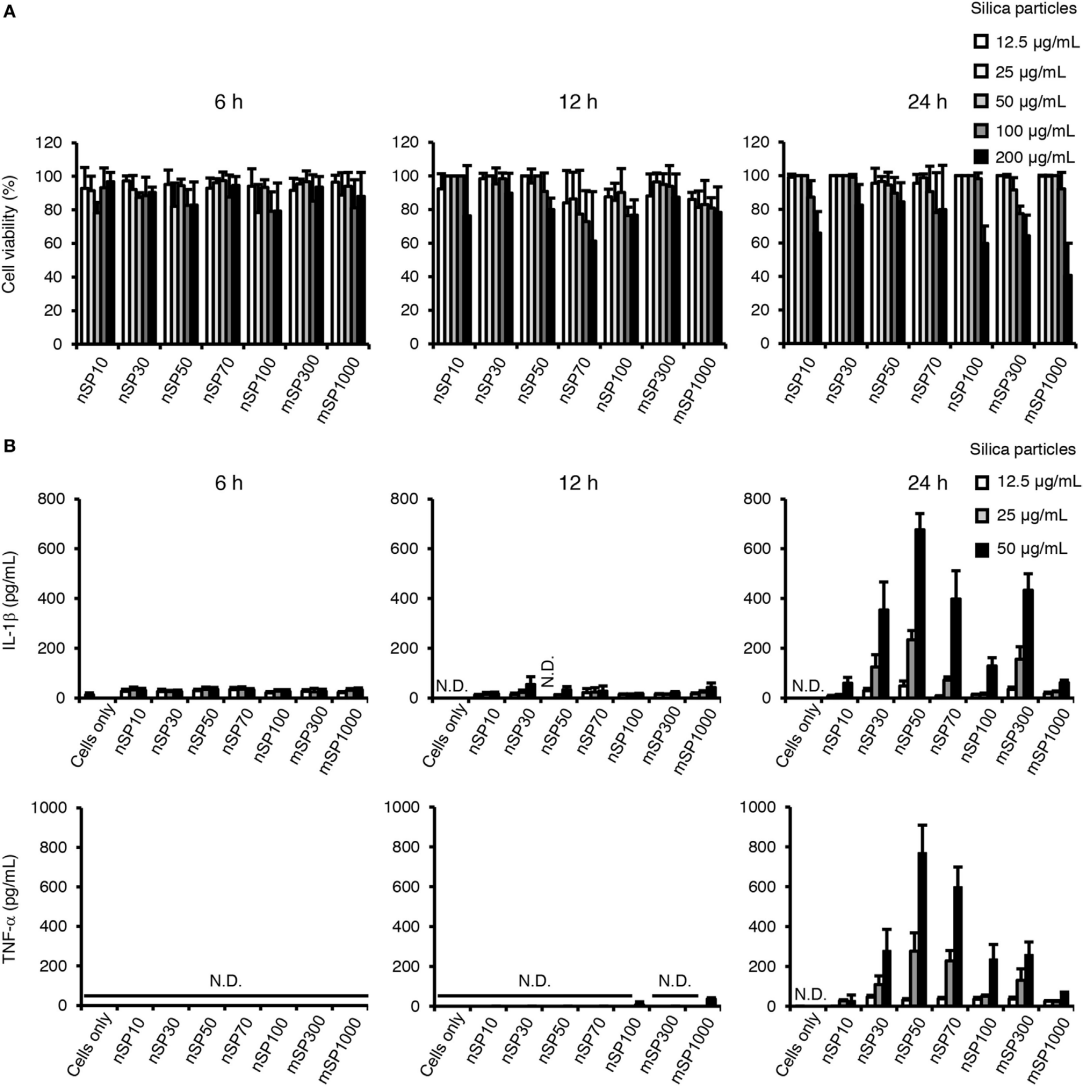
**Effects of silica particles on THP-1 cell viability and pro-inflammatory cytokine secretion**. Phorbol 12-myristate 13-acetate-differentiated THP-1 cells were incubated with silica particles of various concentrations for 6, 12, or 24 h. After incubation, culture supernatants were collected. **(A)** Cell viability was determined by means of a lactate dehydrogenase assay. **(B)** Interleukin (IL)-1β and tumor necrosis factor (TNF)-α concentration in the culture supernatant was measured by ELISA. Data are presented as mean ± SD (*n* = 4 independent cultures/group). N.D., not detected.

We first evaluated the cytotoxicity of the silica particles in THP-1 cells by means of a lactate dehydrogenase cytotoxicity assay (Figure 1A). PMA-differentiated THP-1 cells were incubated with the different silica particles (nSP10, nSP30, nSP50, nSP70, nSP100, mSP300, or mSP1000) for 6, 12, or 24 h. We hardly detected cytotoxicity in our dose range at 6 and 12 h. On the other hand, dose-dependent cytotoxicity was observed at a dose greater than 100 μg/mL in all of the silica particle-treated groups at 24 h and the data suggested that larger particles tended to induce stronger cytotoxicity. In the following assays, 50 μg/mL was the maximum dose of silica particles used to avoid inducing cytotoxicity.

To examine the effect of particle size on the pro-inflammatory effects of the silica particles in THP-1 cells, we measured the concentration of the pro-inflammatory cytokines IL-1β and TNF-α in the culture supernatant after incubation of the cells with the silica particles for 6, 12, or 24 h (Figure 1B). Although incubation with the silica particles for 6 or 12 h had little effect on the secretion of IL-1β and TNF-α, incubation for 24 h resulted in a marked increase in the concentrations of IL-1β and TNF-α in the supernatant in several of the silica particle-treated groups. Furthermore, a bell-shaped size-specific effect was observed, where the silica particles with a diameter of 50 nm induced the greatest secretion of IL-1β and TNF-α and silica particles with smaller or larger diameters had progressively less effect (overall size range, 10–1,000 nm). In addition, the transcript levels of IL-1β and TNF-α were increased 24 h after incubation with nSP50 compared with the control group (Figure S1 in Supplementary Material), which was consistent with the results regarding the secreted proteins. As a positive control, we also exposed the cells to crystalline silica and ATP, which has known pro-inflammatory effects, and found that with this exposure the secretion of IL-1β was increased at the 6, 12, and 24-h time points (Figure S2 in Supplementary Material), which is consistent with a previous report (29). Thus, the size-specific pro-inflammatory effects of silica particles had a relatively slow onset compared with that of crystalline silica as a control particle.

**Figure 2:**
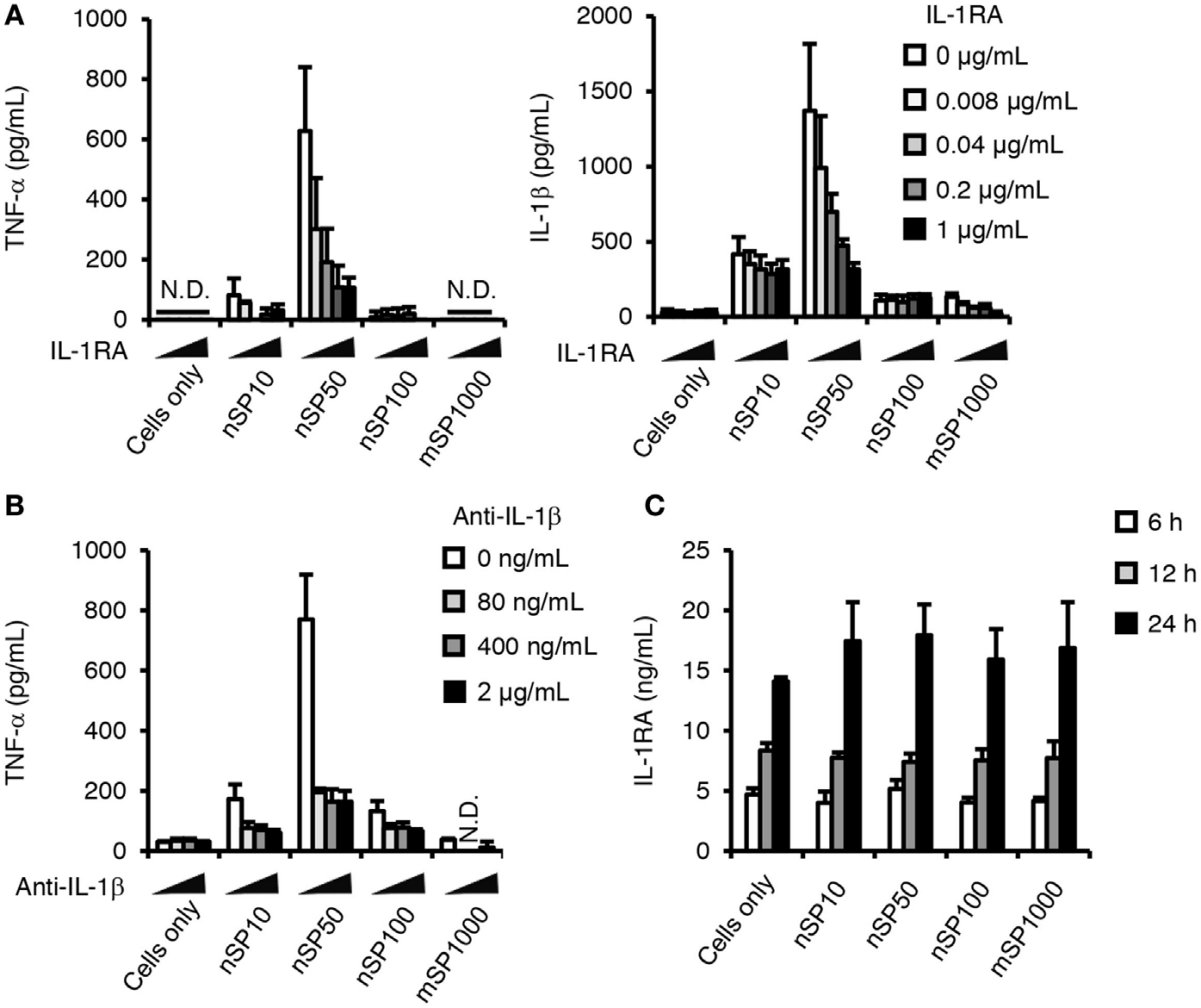
**Role of interleukin (IL)-1β in the pro-inflammatory effects of silica particles**. Phorbol 12-myristate 13-acetate-differentiated THP-1 cells were incubated with IL-1 receptor antagonist (IL-1RA) **(A)** or anti-IL-1β **(B)** 30 min before the addition of silica particles (50 μg/mL). After incubation for 24 h, the concentration of IL-1β **(A,B)** and tumor necrosis factor (TNF)-α **(A)** in the culture supernatant was measured by ELISA. **(C)** Phorbol 12-myristate 13-acetate-differentiated THP-1 cells were incubated with silica particles (50 μg/mL) for 6, 12, or 24 h. After incubation, IL-1RA concentration in the culture supernatants was determined by ELISA. Data are presented as mean ± SD (*n* = 4 independent cultures/group). N.D., not detected.

It has been reported that the induction of TNF-α production by crystalline silica is mediated by IL-1β (29), which implies that the observed increase in TNF-α secretion by exposure to the silica particles may also be meditated by IL-1β. We, therefore, examined the effect of inhibiting IL-1 signaling on the silica particle-induced secretion of TNF-α. Co-incubation of the cells with the silica particles (nSP10, nSP50, nSP100, and mSP1000 as representatives of the size effect) and the RA IL-1RA resulted in a marked reduction in the amount of TNF-α secreted by cells incubated with nSP50 (Figure 2A, left). Similar results were obtained after co-incubation of the cells with the silica particles and anti-IL-1β (Figure 2B). Together, these results suggest that the induction of TNF-α by the silica particles was completely dependent on the production of IL-1β. Furthermore, co-incubation with IL-1RA was found to suppress the secretion of IL-1β in nSP50- or mSP1000-treated cells, but not in nSP10- or nSP100-treated cells, suggesting that a positive feedback loop is created for IL-1β in nSP50- or mSP1000-treated cells (Figure 2A, right). We speculated that perhaps nSP50 inhibited endogenous IL-1RA production, which would enhance the effect of IL-1β. However, the concentration of IL-1RA in the culture supernatants of the cells exposed to the silica particles was comparable with that in the supernatants of unstimulated cells (Figure 2C).

**Figure 3:**
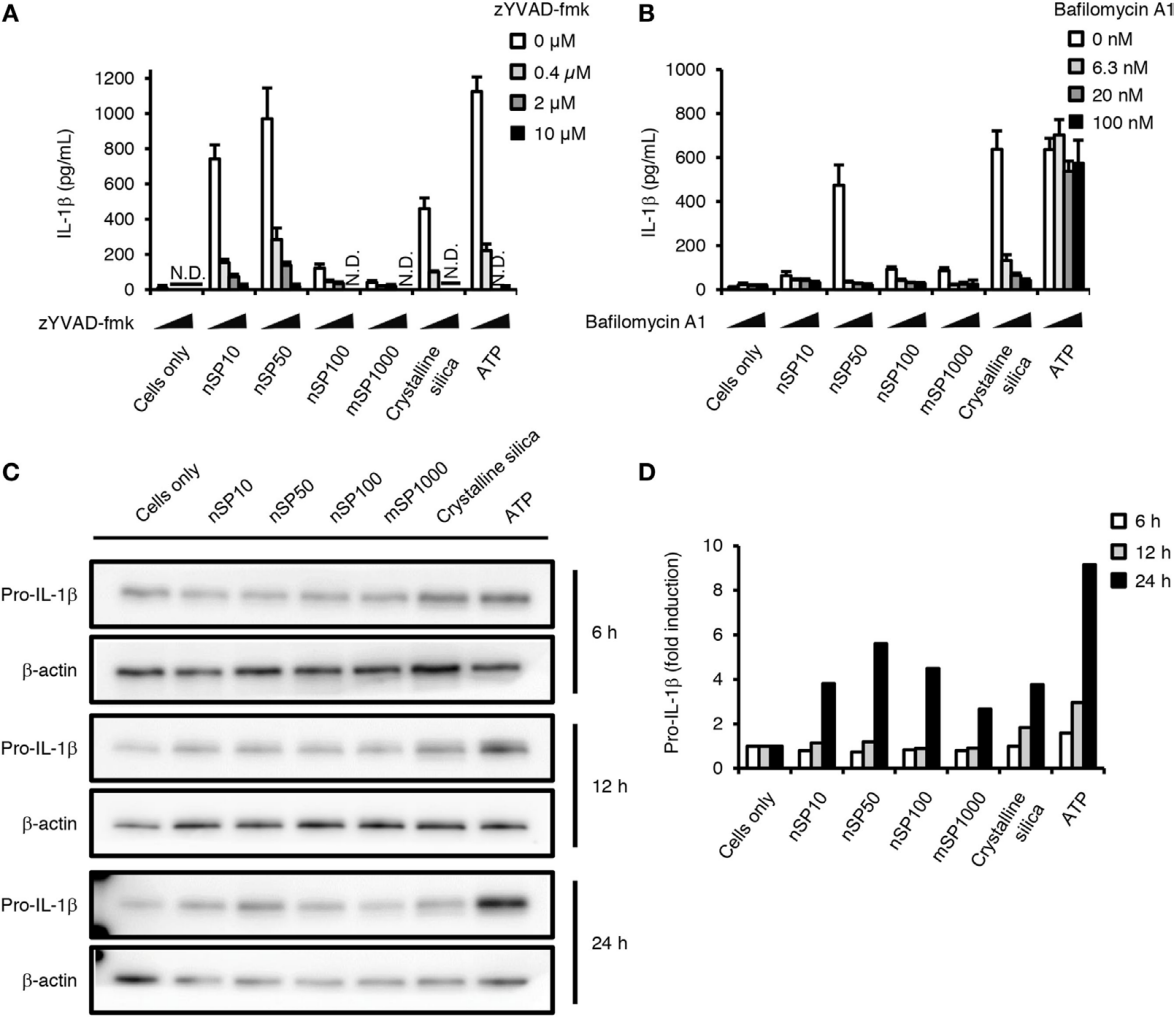
**Effect of silica particles on caspase-1 activation and pro-IL-1β synthesis**. Phorbol 12-myristate 13-acetate-differentiated THP-1 cells were incubated with zYVAD-fmk **(A)** or bafilomycin A1 **(B)** 30 min before the addition of silica particles (50 μg/mL), crystalline silica (500 μg/mL), or adenosine 5′-triphosphate disodium salt hydrate (ATP, 3 mM). Twenty-four hours after incubation, culture supernatants were collected and interleukin (IL)-1β secretion was determined by ELISA. Data are presented as mean ± SD (*n* = 4 independent cultures/group). N.D., not detected. **(C)** Phorbol 12-myristate 13-acetate-differentiated THP-1 cells were incubated with silica particles (50 μg/mL), crystalline silica (500 μg/mL), or ATP (3 mM) for 6, 12, or 24 h, and pro-IL-1β in the cells was analyzed by means of western blotting. **(D)** The densities of the bands of pro-IL-1β in **(C)** were quantified by using the ImageJ software (version 1.46r, National Institutes of Health). The values of pro-IL-1β were normalized to that of β-actin. Fold induction is relative to the cells only group.

Two processes are involved in the secretion of mature IL-1β: NF-κB-dependent pro-IL-1β synthesis and NLRP3 inflammasome (caspase-1)-dependent cleavage of pro-IL-1β (30). Therefore, next we evaluated the effect of exposure to the silica particles on these two processes. Blocking the maturation of IL-1β with zYVAD-fmk, a caspase-1 inhibitor, considerably reduced the concentration of IL-1β in the culture supernatants of the silica particle-treated cells and in the crystalline silica- or ATP-treated cells, which were positive controls for activation of the NLRP3 inflammasome (30) (Figure 3A). These results suggest that induction of IL-1β by silica particles is dependent on the NLRP3 inflammasome. In addition, flow cytometric evaluation of the binding of a fluorescence-coupled YVAD inhibitor of caspase-1 activation showed that caspase-1 tended to be activated in cells treated with nSP10, nSP50, nSP100, or mSP1000 (Figure S3A in Supplementary Material).

Particulate matter such as crystalline silica and alum is known to activate the NLRP3 inflammasome *via* lysosomal destabilization, and neutralization of lysosomal pH inhibits this activation pathway (30). In the present study, inhibiting lysosomal acidification by co-incubation with bafilomycin A1, an inhibitor of vacuolar-type H^+^-ATPase, reduced the induction of IL-1β by all of the silica particles or crystalline silica, but not that induced by ATP (Figure 3B), which is independent of lysosomal destabilization (31). Furthermore, we found that loss of the red acidity-dependent acridine orange signal, which is an index of lysosomal integrity, was significantly increased in nSP50- or crystalline silica-treated cells, but not in ATP-treated cells (Figure S2B in Supplementary Material). The loss of the red acidity-dependent acridine orange signal appeared to be enhanced in nSP10- or nSP100-treated cells (Figure S3B in Supplementary Material). These findings suggest that, like crystalline silica, silica particles activate the NLRP3 inflammasome *via* lysosomal destabilization (32). Thus, the present results show that the silica particles induced the secretion of IL-1β *via* activation of the NLRP3 inflammasome. The results imply that nSP50 activated the NLRP3 inflammasome more than did the other sizes of silica particles *via* stronger induction of lysosomal destabilization (Figure S3 in Supplementary Material).

We next examined the effects of silica particle size on the induction of pro-IL-1β. Since we detected pro-IL-1β in untreated cells, PMA-differentiation has induced a certain amount of pro-IL-1β, which is consistent with a previous report (Figures 3C,D) (32). The expression of pro-IL-1β was increased in cells incubated with ATP for 12 or 24 h (Figures 3C,D). In addition, nSP50 induced more pro-IL-1β compared with the other silica particles after incubation for 24 h (Figures 3C,D). This induction of pro-IL-1β further confirms that a positive feedback loop for IL-1β is created in nSP50-treated cells (Figure 2A, 24 h).

### Relationship between Cellular Uptake and the Size-Specific Pro-inflammatory Effect of Silica Particles

**Figure 4:**
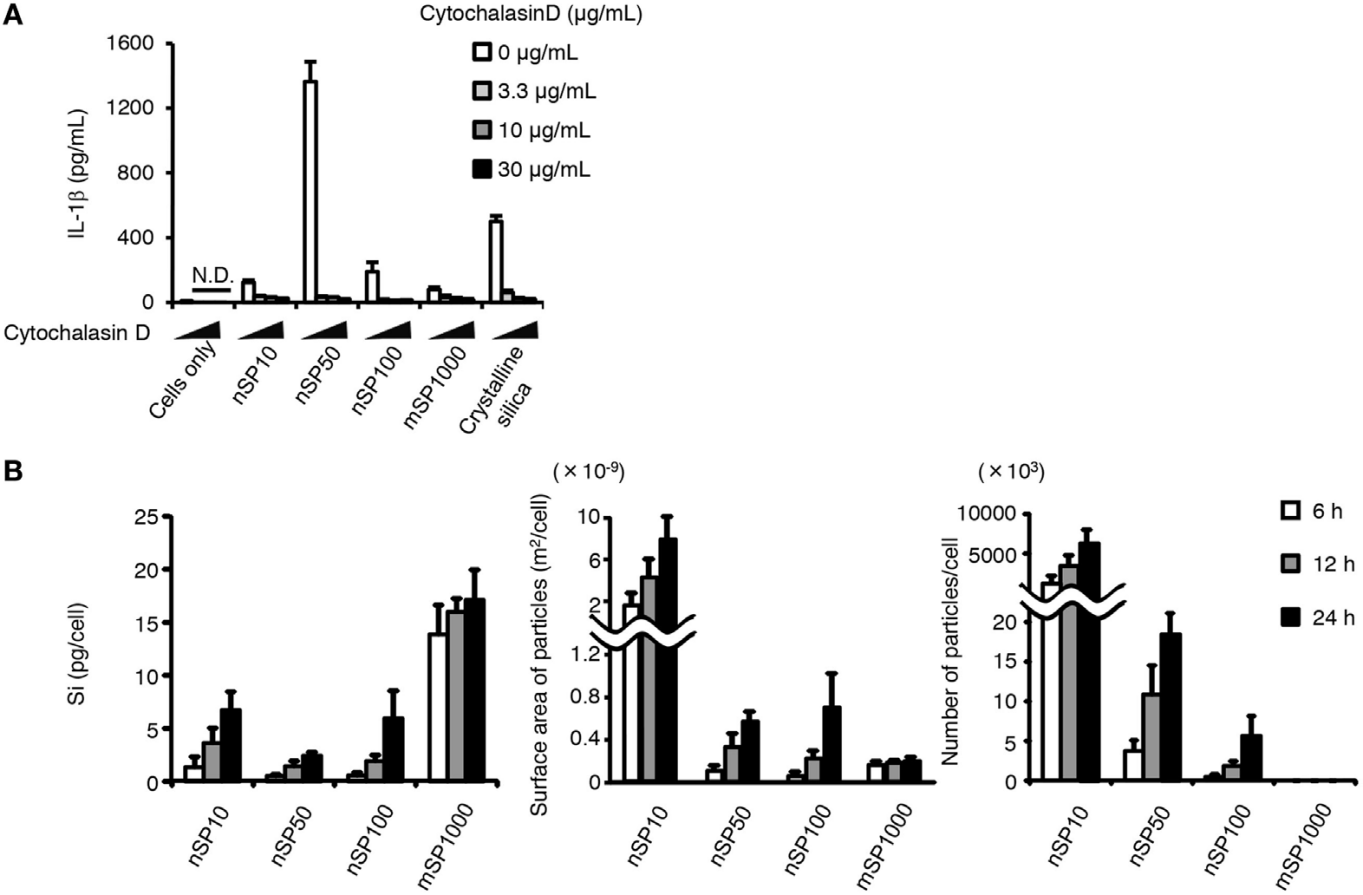
**Relationship between uptake of silica particles and interleukin (IL)-1β secretion**. **(A)** Phorbol 12-myristate 13-acetate-differentiated THP-1 cells were incubated with cytochalasin D 30 min before the addition of silica particles (50 μg/mL) or crystalline silica (500 μg/mL). Twenty-four hours after incubation, culture supernatants were collected and IL-1β secretion was determined by ELISA. **(B)** Phorbol 12-myristate 13-acetate-differentiated THP-1 cells were incubated with silica particles (50 μg/mL) for 6, 12, or 24 h. The amount of silicon in the cells was then determined by inductively coupled plasma atomic emission spectrometry. Silicon uptake by the cells was calculated as the amount of silicon in silica particle-treated cells minus the amount of silicon in non-treated cells. The total particle surface area and number of particles taken up by cells was calculated from information provided in the manufacturer’s data sheet for each type of silica particle. Data are presented as mean ± SD (*n* = 4 independent cultures/group). N.D., not detected.

It is well known that endocytosis of particulate matter triggers the pro-inflammatory responses. We, therefore, evaluated whether the size-specific pro-inflammatory effect of silica particles was endocytosis dependent. Blocking actin-dependent endocytosis with cytochalasin D, a potent inhibitor of actin polymerization, completely suppressed the induction of IL-1β in the silica particle- or crystalline silica-treated cells (Figure 4A). This result suggests that the induction of IL-1β by the silica particles or crystalline silica was dependent on actin-dependent endocytosis. Therefore, we hypothesized that the size-specific pro-inflammatory effects of the silica particles were a result of greater uptake of nSP50 than of the other sizes of silica particles. We, therefore, quantitatively measured by means of ICP-AES the amount of silicon inside cells exposed to the silica particles. A time-dependent increase in the uptake of silica particles was observed in all of the silica particle-treated cells (Figure 4B). The greatest concentration of silicon was found in the cells treated with mSP1000, whereas the least was found in the cells treated with nSP50 (Figure 4B, left panel). By using the concentration of silicon in the cells to calculate the surface area and number of silica particles taken up, we found that nSP10-treated cells contained the greatest total particle surface area and number of silica particles (Figure 4B, center and right panels). Therefore, the induction of IL-1β by the silica particles was not correlated with the total mass of silicon, the total particle surface area, or the number of silica particles taken up by the cells, even though IL-1β secretion appeared to be completely dependent on the uptake of the silica particles *via* actin-dependent endocytosis. We, therefore, hypothesized that a specific mode of endocytosis that is only used for the uptake of silica particles in a specific size range was responsible for their observed size-specific pro-inflammatory effect.

**Figure 5:**
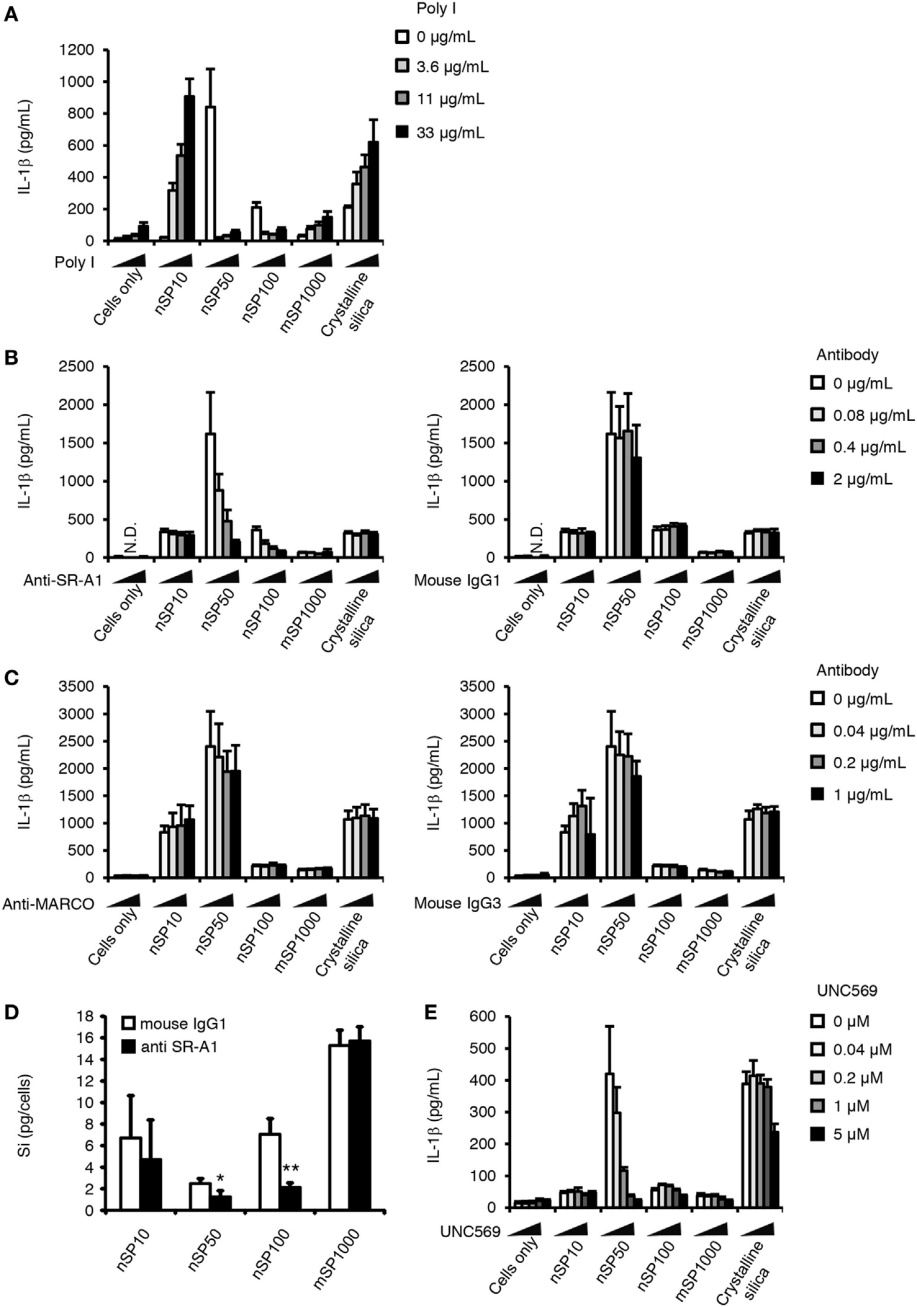
**Role of scavenger receptor (SR)-A1 and Mer receptor tyrosine kinase (MerTK) in the size-specific pro-inflammatory effects of silica particles**. Phorbol 12-myristate 13-acetate-differentiated THP-1 cells were incubated with poly I **(A)**, anti-SR-A1 or its isotype control (mouse IgG1) **(B,D)**, anti-MARCO antibody or its isotype control (mouse IgG3) **(C)**, or UNC569 (a specific inhibitor of MerTK) **(E)** 30 min before the addition of silica particles (50 μg/mL) or crystalline silica (500 μg/mL). **(A–C,E)** Twenty-four hours after incubation, culture supernatants were collected and the concentration of interleukin (IL)-1β was determined by ELISA. **(D)** The amount of silicon in the cells was determined by inductively coupled plasma atomic emission spectrometry. Silicon uptake by the cells was calculated as the amount of silicon in treated cells minus the amount of silicon in non-treated cells. Data are presented as mean ± SD (*n* = 4 independent cultures/group). *P < 0.05, **P < 0.01 versus isotype control group. N.D., not detected.

Class A scavenging receptors (SR-A) are a group of receptors reported to be involved in the uptake into cells of environmental particles, including artificial nanoparticles such as amorphous silica nanoparticles (33, 34). Therefore, we examined the effect of poly I, a scavenging receptor antagonist, on the induction of IL-1β. Poly I treatment enhanced the induction of IL-1β secretion in nSP10-, mSP1000-, or crystalline silica-treated cells, but markedly suppressed it in nSP50- or nSP100-treated cells (Figure 5A). Since poly I is reported to have inflammatory potential as a ligand of toll-like receptor 3 (35), one explanation for this observation in poly I-treated cells is that the uptake of nSP50 and nSP100 was blocked by poly I, the uptake of nSP10, mSP1000, and crystalline silica was completely independent of SR-A, and the inflammatory potential of poly I enhanced IL-1β by nSP10, mSP1000, and crystalline silica. To confirm this hypothesis, we examined the effect of neutralizing SR-As, namely SR-A1 or MARCO, which is reported to be endocytic receptors for particulate matter, on the size-specific pro-inflammatory effects of the silica particles (33, 34). Neutralization of SR-A1 suppressed the induction of IL-1β in nSP50- or nSP100-, but not in nSP10-, mSP1000-, or crystalline silica-treated cells (Figure 5B). Neutralization of MARCO did not affect the induction of IL-1β by the silica particles or crystalline silica (Figure 5C). Neutralization of SR-A1 also significantly reduced the uptake of nSP50 (*P* < 0.05) and nSP100 (*P* < 0.01), but not of nSP10, mSP1000, or crystalline silica (Figure 5D). Thus, it is likely that the size-specific pro-inflammatory effect of the silica particles was a result of SR-A1-mediated endocytosis of particles in a specific size range.

A remaining question is why exposure to nSP50 had a greater effect on the induction of IL-1β than exposure to nSP100 even though both appeared to be taken up *via* the same receptor. It is known that SR-A1 lacks enzymatic activity and intracellular signaling motifs, and that it induces Mer receptor tyrosine kinase (MerTK) signaling (36). Therefore, we evaluated the contribution of MerTK to the induction of IL-1β by nSP50 or nSP100 by using UNC569, an inhibitor of the phosphorylation of MerTK (37). Inhibition of MerTK signaling markedly suppressed the induction of IL-1β by nSP50, but not by nSP100 (Figure 5E). Consistent with the effect of neutralizing SR-A1, the induction of IL-1β by nSP10-, mSP1000-, or crystalline silica was not blocked by UNC569. Together, these results suggest that although the uptake of both nSP50 and nSP100 was dependent on SR-A1, only nSP50 appeared to induce MerTK signaling, which in turn produced a greater induction of IL-1β.

## Discussion

The present results suggest that SR-A1-mediated endocytosis underlies silica particle-induced IL-1β secretion, and that the size-specific pro-inflammatory effects of silica particles are a result of the ligand size specificity of this SR-A1-mediated endocytosis. The present results also suggest that silica particles with a diameter of 50 nm induced the strongest pro-inflammatory response. SR-A1 is known to mediate both pro- and anti-inflammatory responses due to its broad ligand specificity (38). However, it remains unknown how different ligands produce opposite responses *via* the same receptor. One report has demonstrated that SR-A-mediated ligand endocytosis is mediated *via* clathrin-dependent and caveolae-dependent pathways, and that each endocytic mode has distinct functional consequences *via* different signaling cascades (39). Interestingly, clathrin-mediated and caveolae-mediated endocytosis are known to be limited to ligands with sizes of about 120 and 60 nm, respectively (40). Given these size limitations, in the present study, only nSP100 or smaller particles could be taken up *via* clathrin-mediated endocytosis, and only nSP50 (or possibly nSP70) or smaller silica particles could be taken up by caveolae-mediated endocytosis. Therefore, it is possible that the ligand size limit of each endocytic mode contributed to the particle size-specific effects. Further studies are required to elucidate the relationship between each endocytic mode of SR-A and the activation of MerTK, which in turn produced the greater pro-inflammatory effects.

The results of the present study also suggest that the uptake of nSP10 is independent of SR-A1-mediated endocytosis (Figure 5D). Small nanoparticles are suggested to be difficult to promote multivalent binding by the receptors and thus smaller nanoparticles dissociate from the receptors before being taken up by cells due to low binding avidity (18). Therefore, it is possible that low avidity of nSP10 to SR-A1 cause the independency of SR-A1-mediated endocytosis. In addition, for silica particles to be ligands of SR-A1 they must be anionic in some extent; therefore, the number of silanol groups on the surface of silica particles will determine whether or not they are ligands of SR-A1. Since, the concentration of silanol groups on the surface of silica nanoparticles increases as particle size decreases (41), the ability of the silica particles to bind to SR-A1 must be changed dependent on the size. Thus it is also possible that nSP10 is not a ligand of SR-A1 due to the too much concentration of silanol groups.

The present results suggest that nSP50-mediated MerTK signaling increased the induction of IL-1β, although MerTK signaling itself is often discussed in an anti-inflammatory, immunosuppressive context mainly due to its relationship to the uptake of apoptotic cells by macrophages (42, 43). It has been reported that MerTK activation leads to inhibition of the mTOR pathway and SR-A1-mediated activation of macrophages (44). Inhibition of the mTOR pathway enhances the effects of pro-inflammatory cytokines *via* NF-κB in phagocytic cells after bacterial stimulation (45) and of caspase-1 during endotoxin-mediated shock (46). In addition, phagocytosis of autophagic dying cells is reported to activate the NLRP3 inflammasome rather than inhibit immune reactions (47). Thus, in our model, MerTK signaling may induce pro-IL-1β and caspase-1 activation, which in turn increases silica particle-induced IL-1β secretion. Further studies are required to determine how MerTK signaling led to the inflammatory state in our model and what phenotype the size-specific effects of silica particles result in *in vivo*.

In previous studies, we observed greater induction of IL-1β in THP-1 cells treated with mSP1000 than in those treated with smaller particles (i.e., nSP30, nSP50, nSP70, mSP300, and mSP1000), although higher concentrations of silica particles were used than in the present study (i.e., 100 μg/mL; 6 h incubation) (48). However, under the present low-cytotoxic conditions, treatment with mSP1000 induced little IL-1β, even after incubation for 24 h (Figure 1). Furthermore, under the present conditions, IL-1β was detected only at 24 h, irrespective of which silica particle the cells were exposed to. Another group has reported that exposure to high concentrations (125–500 μg/mL) of silica nanoparticles with a diameter of 15 nm induced IL-1β after incubation for 6 h, and that active ATP release was the underlying mechanism (26). Interestingly, larger silica particles have also been show to produce greater IL-1β induction (4) that is consistent with the results of our previous study, where we used high doses of silica particles (48). Therefore, it is possible that larger silica particles induce greater production of IL-1β than do smaller silica particles under high-dose (i.e., high-stress) conditions *via* an active ATP release mechanism. Since active ATP release has been shown to be the mechanism underlying the induction of IL-1β by other particles (e.g., uric acid, crystalline silica, alum) (Figure S2 in Supplementary Material, 6 h incubation) (49), it may have evolved to enable cells to rapidly secrete IL-1β. Therefore, it is likely that there are two different mechanisms underlying the induction of IL-1β by silica particles that are activated only during exposure to specific concentrations of silica particles. Further studies are required to elucidate whether the slower SR-A1-mediated IL-1β secretion observed in the present study involves active ATP release.

Scavenger receptors are a potentially useful target for vaccines for vaccine development (50, 51). Although elucidation of the relationship between the SR-A1-mediated size-specific effects of nanoparticles and adjuvanticity is required, optimizing the size of the nanoparticles may be a useful way to maximize the effects of nanoparticle-mediated vaccines. However, the size-specific effects of nanoparticles mean that it is difficult to reliably predict the safety of nanoparticles and so individual safety assessments will likely be required for each new nanoparticle-based product. It is, therefore, important to improve our understanding of the size-specific effects of nanoparticles.

The results of the present study suggest that SR-A1-mediated uptake of nanoparticles led to a size-specific inflammatory response in THP-1 cells. Since nanoparticles also have size-specific effects in non-phagocytic cells (17–19), there are likely additional underlying mechanisms in these cells. Further studies to examine the effects of silica and other nanoparticles on a variety of cell types would improve our understanding of the size-specific effects of nanoparticles. In the present study, nanoparticles with a diameter of around 50 nm were found to have the greatest pro-inflammatory effects, and the size-specific effects of nanoparticles of this size are well reported in the literature. Therefore, further examination not only of the size-specific effects of nanoparticles but also of the possibility that 50 nm is a size that has special implications in biological systems in general is needed.

## Author Contributions

NN, TH, and YY designed the experiments and interpreted the results. NN, TH, KM, and MA performed the experiments and analyzed the data. NN, TH, and YY wrote the manuscript; EK, KI, and KH provided technical support and conceptual advice. YT supervised the project. All authors have read, discussed, and approved the final manuscript.

## Conflict of Interest Statement

YY is employed by the Research Foundation for Microbial Diseases of Osaka University. All other authors declare no competing financial interests.

## Abbreviations

nSP10, nSP30, nSP50, nSP70, nSP100, mSP300, and mSP1000, amorphous silica particles with diameters of 10, 30, 50, 70, 100, 300, and 1,000 nm, respectively; silica particles, amorphous silica particles; crystalline silica, crystalline silica particles; PMA, phorbol 12-myristate 13-acetate; poly I, polyinosinic acid potassium salt; ATP, adenosine 5′-triphosphate disodium salt hydrate; ICP-AES, inductively coupled plasma atomic emission spectrometry; FLICA, fluorescent-coupled YVAD inhibitor to the activated form of caspase-1; SR, scavenger receptor; MARCO, macrophage receptor with collagenous structure.
