# Supplementary material for: Human Scavenger Receptor A1-Mediated Inflammatory Response to Silica Particle Exposure Is Size Specific
Source: Front Immunol. 2017 Apr 3;8:379. doi: 10.3389/fimmu.2017.00379 (PMC5377922; doi:10.3389/fimmu.2017.00379)
Supplement: Supplementary file 1 [file Presentation_1.PDF]

Silica particles	Hydrodynamic diameter (nm)[a]
**nSP10**	10.0 ± 3.0
**nSP30**	24.3 ± 3.5
**nSP50**	48.3 ± 10.6
**nSP70**	64.7 ± 5.8
**nSP100**	86.0 ± 32.7
**mSP300**	285.7 ± 61.5
**mSP1000**	1164.3 ± 101.0

Data are presented as mean ± SD (n = 3).
[a]Peak size from histogram of relative particle number.

Supplementary Table 1 | Hydrodynamic diameters of the silica nanoparticles (5 mg/mL) in the cell culture medium of THP-1 cells.

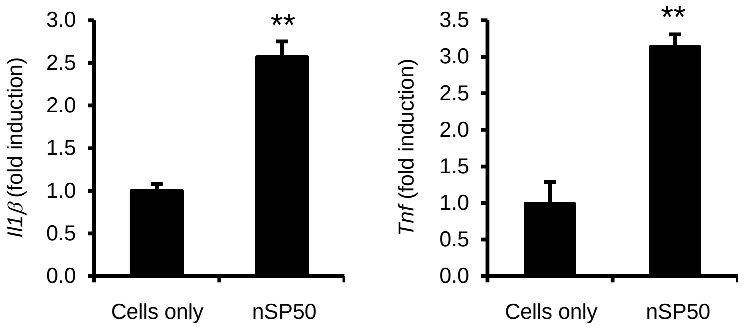

Supplementary Figure 1 | Quantitative evaluation of transcripts of IL-1β and TNF-α in nSP50-treated cells. Phorbol 12-myristate 13-acetate-differentiated THP-1 cells were incubated with nSP50 (50 μg/mL) for 24 h. After incubation, the transcript levels of IL-1β and TNF-α in the cells were determined by means of quantitative PCR and normalized to that of *Gapdh*. Fold induction is relative to the cells only group. Data are presented as mean ± SD (*n* = 5 independent cultures/group; ***P* < 0.01 versus the cells only group).

Supplementary Figure 1. NISHIJIMA et al.

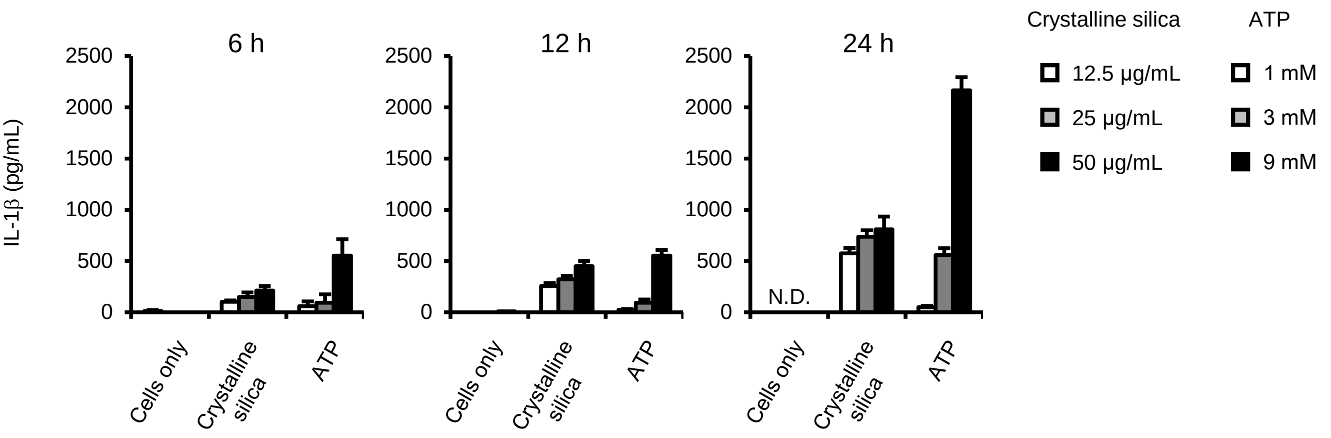

Supplementary Figure 2 | IL-1β secretion induced by exposure to crystalline silica or adenosine 5'-triphosphate disodium salt (ATP) in our model. Phorbol 12-myristate 13-acetate-differentiated THP-1 cells were incubated with crystalline silica or ATP at the indicated concentrations for 6, 12, or 24 h. After incubation, the concentration of IL-1β in the culture supernatant was measured by means of an enzyme-linked immunosorbent assay. Data are presented as mean ± SD (*n* = 4 independent cultures/group). N.D., not detected.

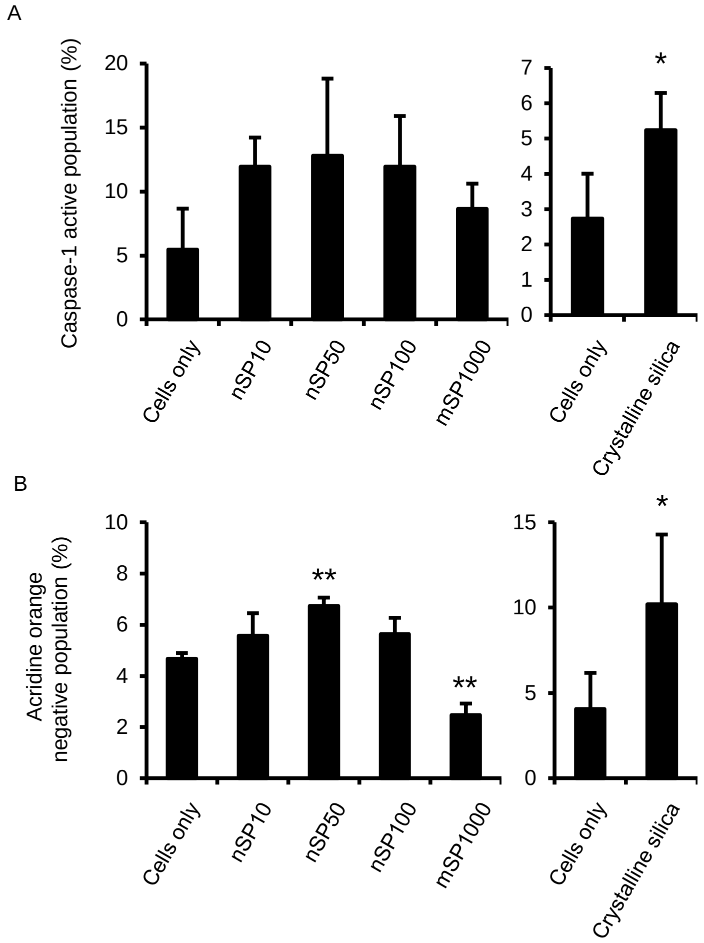

Supplementary Figure 3 | Effect of silica particle size on caspase-1 activation and phagosomal destabilization. Phorbol 12-myristate 13-acetate-differentiated THP-1 cells were incubated with silica particles (50 µg/mL) for 24 h or crystalline silica (500 µg/mL) for 6 h. **(A)** The cells were then stained with a fluorescent inhibitor of caspase-1 activation (FLICA). FLICA-positive cells were considered to be the caspase-1 active population. **(B)** Cells were stained with acridine orange. Lysosomal rupture was assessed by means of flow cytometry as loss of emission at 665–685 nm (excitation, 488 nm). Data are expressed as mean ± SD (n = 3 independent cultures/group; **$P < 0.01$, *$P < 0.05$ versus the cells only group).

Supplementary Figure 3. NISHIJIMA et al.

Supplemental methods

Measurement of hydrodynamic diameter of silica particles

Silica particles were diluted to 5 mg/mL in the cell culture medium of THP-1 cells (see MATERIALS AND METHODS in the main manuscript), and the hydrodynamic diameters were measured by using a Zetasizer Nano ZS90 instrument (Malvern Instruments, Worcestershire, UK) immediately after sonication at 400 W for 5 min and vortex for 1 min.

Quantitative PCR

THP-1 cells (9.0×10^5 cells/well) were seeded in 6-well plates (Nunc, Rochester, NY) and then differentiated into macrophages by incubation with 0.5 μM phorbol 12-myristate 13-acetate at 37°C for 24 h. After incubation, the cells were washed with the cell culture media and treated with nSP50 (50 μg/mL). After incubation for 24 h, total RNA from the cells was extracted by using an RNeasy Mini Kit (Qiagen, Valencia, CA) in accordance with the manufacturer's instructions, and then reverse-transcribed to single-strand complementary DNA (cDNA) by using a SuperScript IV First-Strand Synthesis System (Invitrogen, Carlsbad, CA) with random hexamer primers in accordance with the manufacturer's instructions. Gene-specific primer pairs [*Il1b*: Fwd: 5'-tacctgtcctgcgtgttgaa-3', Rev: 5'-tctttgggtaatttttgggatct-3'; *Tnf*: Fwd: 5'-cagcctcttctccttcctgat-3', Rev: 5'-gccagagggctgattagaga-3'; *Gapdh* (glyceraldehyde phosphate dehydrogenase): Fwd: 5'-agccacatcgctcagacac-3', Rev: 5'-gcccaatacgaccaaatcc-3'] and TaqMan® probes (Universal Probe Library, Roche Applied Science, *Il1b*: #78; *Tnf*: #29; *Gapdh*: #60) were used. Quantitative PCR was performed by using a LightCycler 480 II instrument and LightCycler 480 Probe Master (Roche Applied Science, Penzberg, Germany), and values were normalized to the expression of *Gapdh* mRNA. The expression level of each mRNA is shown as a relative fold-change. Relative expression was compared between the nSP50-treated group and the untreated group.

Evaluation of lysosomal destabilization

To evaluate lysosomal rupture, phorbol 12-myristate 13-acetate-differentiated THP-1 cells were incubated with the silica particles (50 μg/mL) for 24 h. After incubation, the cells were washed two times with the cell culture media and incubated for a further 15 min with acridine orange (0.2 mg/mL). Lysosomal rupture was assessed by means of flow cytometry as loss of emission at 665–685 nm (excitation, 488 nm). An LSRFortessa cell analyzer (BD Biosciences, San Jose, CA) was used for all flow cytometry. Data were acquired with the DIVA software (BD Biosciences).

Caspase-1 activity assay

Caspase-1 activity in phorbol 12-myristate 13-acetate-differentiated THP-1 cells was determined by using a Caspase-1 FLICA kit (Immunochemistry Technologies, Bloomington, MN) in accordance with the manufacturer's instructions. Briefly, the differentiated THP-1 cells were incubated with the silica particles (50 μg/mL) for 24 h. After incubation, the cells were washed two times and then trypsynized. FAM-YVAD-fmk, a FAM-FLICA caspase-1 reagent, was reconstituted with dimethyl sulfoxide and diluted with phosphate buffered saline to a concentration of 150 mM. FAM-YVAD-fmk was added to the cultured media including the cells to a final concentration of 5 mM. The cells were incubated for 45 minutes at 37°C. Caspase 1 activity was assessed by means of flow cytometry as loss of emission at 515–545 nm (excitation, 488 nm). An LSRFortessa cell analyzer (BD Biosciences) was used for all flow cytometry. Data were acquired with the DIVA software (BD Biosciences).
